# 519. Modulation of Post-burn Pulmonary Dysfunction via Novel TIE2 Agonist

**DOI:** 10.1093/jbcr/irag033.178

**Published:** 2026-04-06

**Authors:** Natalia Carbajal Garcia, Mary Grace Murray, Mashkoor A Choudhry, John Kubasiak

**Affiliations:** Burn and Shock Trauma Research Institute, Loyola University Chicago, Oak Park, IL; Loyola University Medical Center, Maywood, IL; Loyola University Medical Center, Maywood, IL; Loyola University Medical Center, Maywood, IL

## Abstract

**Introduction:**

Angiopoietin-2 (Ang-2), an antagonist of TIE2 is a key mediator of capillary permeability and vascular leak, particularly in sepsis and acute respiratory distress syndrome (ARDS), a complication of post-burn state. We recently identified an early increase in Ang-2 following burn predicts subsequent 30-day development of pneumonia in patients. In a murine model of burn injury, there is increased plasma Ang-2 which correlates with pulmonary congestion, inflammation, neutrophil infiltration and tissue edema. As leaky microvessels promote tissue edema and systemic loss of intravascular fluid, these features highlight vascular-barrier stabilization as a modern therapeutic objective. AV-001 is a polyethylene glycol (PEG)-clustered Tie2 agonist peptide.

In this study, we used a murine burn model to define the lung response to AV-001. We quantified cytokines and characterized immune-cell infiltration within the lung microenvironment.

**Methods:**

8–10-week-old male C57/BL6 mice (Charles River) were subjected to sham injury or 12.5% TBSA scald burns. Mice were treated with saline plus placebo, low (17.5 ug) or high-dose (35 ug) of AV-001. One day post-injury, blood was collected via cardiac puncture, and lungs were collected. Heparinized plasma and lung homogenates were profiled using the Mouse Angiogenesis & Growth Factor 16-Plex Discovery Assay® (Eve Technologies). Group differences were tested by ordinary one-way ANOVA in GraphPad Prism. Lung sections were inflated and fixed in formalin for histology, stained with hematoxylin–eosin (H&E), and quantified in QuPath and ImageJ for immune-cell infiltration, alveolar airspace, and pneumocyte counts.

**Results:**

We found an increase in lung Ang-2 levels after burn injury compared to shams, this further confirms our previous observations (Fig. 1. 1A, *p*=.13). Among the molecules measured, the most prominent differences were detected for EGF in plasma (Fig. 1B *p*=.008) and lung VEFG-A (Fig. 1C *p* = < 0.001) in which we observed a significant return toward baseline with the low dose. In the quantified data in QuPath and ImageJ we found prominent differences in alveolar airspace (Fig. 2. 2A, *p* = < 0.0001) and total cellularity (Fig. 2. 2B, *p* = *p* = < 0.0001) with a significant improvement with the low dose.

**Conclusions:**

These findings suggest that AV-001 at low-dose treatment significantly shifted values toward baseline, given that both molecules are pivotal growth factors involved in the development, maintenance and disease reaction: EGF aiding in fluid clearance and tissue repair after injury and VEFG-A promoting the repair of the alveolar-capillary membrane, their modulation may have therapeutic implications. Further studies are necessary to understand if modulating TIE2 signaling can protect against pneumonia and ARDS post-burn.

**Applicability of Research to Practice:**

This research explores a new therapeutic agent targeting Tie2 in the context of burn.

**Funding for the study:**

This work was supported by the National Institutes of Health: [R35GM160273].

